# Factors associated with recent intimate partner violence experience amongst currently married women in Afghanistan and health impacts of IPV: a cross sectional study

**DOI:** 10.1186/s12889-018-5507-5

**Published:** 2018-05-03

**Authors:** Andrew Gibbs, Julienne Corboz, Rachel Jewkes

**Affiliations:** 1 Gender and Health Research Unit, South African Medical Research Council, Pretoria, South Africa; 2 What Works to Prevent Violence Against Women and Girls? Global Programme, Pretoria, South Africa

**Keywords:** Afghanistan, Violence against women, Gender, Intimate partner violence

## Abstract

**Background:**

Intimate partner violence (IPV) is exceedingly common in conflict and post-conflict settings. We first seek to describe factors associated with past 12 month IPV amongst currently married women in Afghanistan, focused on the factors typically assumed to drive IPV. Second, to describe whether IPV is independently associated with a range of health outcomes.

**Methods:**

Cross-sectional analysis of currently married Afghan women, comprising the baseline study of a trial to prevent IPV. We use multinomial regression, reporting adjusted relative-risk ratios to model factors associated with the different forms of IPV, comparing no IPV, emotional IPV only, and physical IPV and emotional IPV. Second we assessed whether experience of emotional IPV, and physical IPV, were independently associated with health outcomes, reporting adjusted ß coefficients and adjusted odds ratios as appropriate.

**Results:**

Nine hundred thirty five currently married women were recruited, 11.8% experienced only emotional IPV and 23.1% experienced physical and emotional IPV. Emotional IPV only was associated with attending a women’s group, greater food insecurity, her husband having more than one wife, experiencing other forms of family violence, and more inequitable community gender norms. Experiencing both physical IPV and emotional IPV was associated with attending a women’s group, more childhood trauma, husband cruelty, her husband having more than one wife, experiencing other forms of family violence, more inequitable community gender norms, and greater reported disability. Emotional IPV and physical IPV were independently associated with worse health outcomes.

**Conclusion:**

IPV remains common in Afghanistan. Economic interventions for women alone are unlikely to prevent IPV and potentially may increase IPV. Economic interventions need to also work with husbands and families, and work to transform community level gender norms.

**Trial registration:**

NCT03236948. Registered 28 July 2017, retrospectively registered.

## Background

Intimate partner violence (IPV) is common in both conflict and post-conflict settings, but there remains little evidence about the extent of IPV in such settings [1, 2]. For four decades, Afghanistan has experienced high levels of conflict. Country wide research suggests about 50% of Afghan women have lifetime IPV experience [3, 4], with wide variation from 6% in Helmand and 7% in Badakhshan Provinces, to 92% in Ghor and Herat Provinces [5]. Estimates of past year IPV are around 30% for the country [4]. Risk factors associated with IPV in Afghanistan include early marriage, gender inequitable attitudes, women’s poverty and low education and acceptability of IPV [4, 6].

Global research on risk factors for IPV have focused on individual characteristics, partner characteristics, poverty, and community attitudes [7]. Individual characteristics include education level and age, and gender attitudes [8, 9]. Increasingly, childhood physical, sexual, and emotional abuse and neglect, are recognized to shape women’s vulnerability to IPV [10, 11]. Women’s poor mental health, including depression and post-traumatic stress disorders (PTSD), are understood as risk factors for IPV [12, 13]. Disability is also a risk factor for IPV, but the majority of studies on these associations come from Europe and north America [14].

Characteristics of women’s male partners are important in shaping women’s vulnerability to IPV. Men who are more controlling, who are more patriarchal in their attitudes and practices [15, 16], and who have more sexual partners and use substances [15] are more likely to perpetrate IPV. Additionally, in Asian countries, the role of mother-in-laws in driving IPV, is also highlighted, with mother-in-laws sometimes encouraging sons to be violent to their wife’s, else perpetrating violence against the daughter-in-law themselves [17–20].

Household economic position is also important in women’s experiences of IPV. Household food insecurity is often a key marker of IPV vulnerability, even in high-income countries [8, 21]. Other markers of household and women’s economic position, such as earnings, savings and assets, have not been so consistent in their associations with IPV vulnerability [8, 22, 23].

Finally, community level analyses have consistently shown that where IPV is normative at the community level, women are more likely to experience IPV [24]. But, in contexts where laws support women’s rights and women’s access to resources, and therefore women’s autonomy, IPV is less common [24].

IPV is associated with women’s poor health. Women who experience IPV are more likely to be depressed, suicidal, have post-traumatic stress disorder (PTSD), and have overall worse health [13, 25]. There are two gaps in the literature on the health impacts of IPV. First, there is little evidence from conflict/post-conflict settings on the health impacts of IPV. The majority of research in conflict settings has focused on the short- and long-term impacts of conflict on health [26], but IPV remains the most common form of violence women experience in conflict settings and it remains important to understand whether IPV has independent health impacts even in contexts of generalized trauma and violence. Second, the health impacts of emotional IPV, independent of physical IPV, is slowly being recognized [27–30], but there remains limited evidence across settings about whether this holds true.

This paper has two aims. First, to describe the factors associated with recent IPV amongst a group of currently married women in Afghanistan, focused on the factors typically assumed to drive IPV. Second, to describe whether IPV is independently associated with health outcomes amongst these married women.

## Methods

Data are drawn from currently married women participating in the baseline assessment of the Women for Women International (WfWI) intervention trial, in Afghanistan, enrolled between September 2016 and March 2017. The study comprised six villages in Kabul and Nangarhar Provinces.

Women were aged 18 to 49. The study aimed to recruit among the poorest women in villages, and those without much formal education, and not currently working or earning much money. In the main study, women were recruited no matter their marital status, but in this analysis only married women are included, as it is not possible to ask about IPV to women who are not currently married.

Women were randomized at the individual level to intervention and control arms, and were aware of arm allocation when completing questionnaires. For questionnaire completion, women in the control arm received US$10, while women in the intervention received no compensation (however they did receive the intervention). The intervention was delivered after baseline data collection, and seeks to strengthen livelihoods and social empowerment [31, 32]. Ethical approval for the study was received from the South African Medical Research Council and the Afghan Ministry of Health. Further information on study procedures are described elsewhere [32].

### Data collection

Structured paper and pencil questionnaires were completed through face-to-face interviews. Fieldworkers were women trained in quantitative interviewing. Interviews were conducted in training centres in villages, a space only women could enter, where auditory privacy could be ensured.

### Data management

Data was double-entered into a database. Discrepancies between the databases were resolved through manual checking of the original questionnaire. Missing data was imputed for scales. The mean of the item for the overall sample was used to impute data and no more than two items were imputed per individual, per scale. If three or more items were missing from a scale for an individual, the scale was set to missing for that person.

### Measures

The primary outcome was past year IPV and only married women were asked these questions. Emotional IPV and physical IPV were assessed using scales based on the WHO Multi-Country study on Domestic Violence [25], adapted and tested in Asia [22]. Both emotional and physical IPV were assessed using behaviourally specific items, with responses to each item either, never, once, few, or many. Five physical IPV questions were asked, which included acts such as whether the woman had been slapped, pushed, hit, threatened with a knife or gun, or had them used on her, and seven emotional IPV, questions were asked, including items about being humiliated, belittled, scared, or threatened.

Socio-demographic questions were asked, including ethnic group, age, marital status and education level. Women were asked a single item about participation in a women’s group, specifically “Do you attend a group where women meet together to do something to earn money or to improve the community or for social reasons in the village?” Women’s gender attitudes were assessed using 11 items, on the gender equity scale, modified for use in Afghanistan (α = 0.87), with a typical item being “I think girls in my family should go to school”. Items were summed together (range 14–32), with higher scores indicating less gender equitable attitudes. Childhood traumas were assessed using 12 items based on the childhood trauma scale [33] adapted for Afghanistan, a typical item was “Before I married I saw or heard my mother being beaten by her mother-in-law or another person in the family”. Items included experiences of physical violence, hunger, and neglect before the age of 18 (α = 0.65). Items were summed (range 12–36) and higher scores indicated more childhood trauma.

Poverty was assessed in two ways. Household food insecurity was assessed with three items of the Household Hunger Scale (α = 0.94), assessing past month food insecurity, which was summed (range 3–12) [34]. One item assessed financial status, asking about past month earnings.

Household relationships were assessed in three different ways. First women were asked whether they were their husband’s only wife. Second, a five-item scale assessed husband cruelty, with questions including “my husband is strict and controlling”. Responses were on a four-point Likert scale and summed (α = 0.88, range 8–14). Finally, to assess violence in the home from other family members, women were asked three questions about violence experienced from other household members; mother-in-law, father, or a sibling, all in the past year. A positive response to any of these items led to women being classified as having experienced violence from another family member.

Perceived community level gender attitudes were assessed using 11 items. Community level gender attitudes were assessed through the same questions as individual level gender attitudes, but instead of “I think”, questions were “In this community many people think” (α = 0.90, range 13–37).

Six health and wellbeing measures were assessed. Past week depression was assessed with CESD-20 [35] and scores were summed (α = 0.90, range 0–54). Functional limitations/disability were assessed through summation of six items (α = 0.63, range 0–15) of the Washington Group Short Set of Disability Questions [36]. Both depression and disability were considered as ‘drivers’ and outcomes of IPV, based on previous literature [12]. Past week PTSD symptoms were assessed using 16 items of the Harvard Trauma Questionnaire [37]. A mean score was calculated (α = 0.92, range 16–62). Satisfaction with life was assessed using four items from the Life Satisfaction scale [38], higher scores indicating less satisfaction (α = 0.90, range 4–20). A single item assessed current overall health on a five point Likert scale; higher scores indicated worse health.

### Analysis

We first describe the socio-demographic characteristics, livelihoods, and experiences of IPV of the sample used for the analysis with percentages and means. As there is significant overlap between the different forms of IPV, we created a three-level categorization; women experiencing no IPV; those experiencing one or more instances of emotional IPV (but no physical IPV); and those who experienced both physical IPV and emotional IPV. Only 1.8% (*n* = 17) had only experienced physical IPV, but not emotional IPV, and were excluded from analysis. For the categorical variables, proportions of the sample with each level of the variable by IPV category are presented. For continuous variables, the mean for each experience category are presented. We used Chi-square tests to compare distributions across categorical variables and present 95% confidence intervals (CIs) to enable comparison of means, as appropriate, by IPV category. All variables were selected based on an a priori hypothesis that they may be associated with IPV, based on previous research on risk factors for IPV, summarized in the introduction.

We used multinomial regression to model factors associated with the different forms of IPV, comparing no IPV experience, one or more experiences of emotional IPV only, and one or more experiences of physical IPV and one or more experiences of emotional IPV. Variable elimination was not used in model building and we include all variables from the descriptive table. We tested for multicollinearity in the model, and found none. To ensure we had enough power in the model we assessed the number of events in the sample, and used the rule of thumb requiring 10 participants per covariate in a regression model [39]. Our sample size and number of events meant we could model up to 14 covariates in the regression.

To estimate health impacts of the different forms of IPV we measured (emotional and physical) we categorsied women’s experience of IPV in two ways. First, we considered the health impacts with IPV categorized as for the above model: no IPV, one or more experiences of emotional IPV only, and one or more experiences of physical IPV and one or more experiences of emotional IPV. In Table 4 these comprise the first three rows (IPV categorization 1). Second, recognizing severity of IPV experience is important for health impacts we created a second three-level variable for women: those who had experienced either none or one physical IPV or none or one emotional IPV experiences; two or more experiences of emotional IPV only; and two or more physical IPV and two or more experiences of emotional IPV (IPV categorization 2). This approach to modelling IPV experienced has been used previously to disentangle whether emotional IPV has an independent health impact on women’s health, aside from physical IPVs [30]. We assessed whether these different categorisations were independently associated depression, PTSD symptoms, life satisfaction, self-reported health, suicidal ideation and disability severity, adjusting for age, education and clustering.

## Results

In total, 935 currently married women were recruited into the evaluation (Table 1). Of these women, 33% reported any emotional IPV in the past year, and 23% reported physical IPV in the past year. In total, 35% reported any physical and/or emotional IPV in the past year. For the ordinal categorization of IPV, 66.2% experienced no IPV, 11.8% experienced only emotional IPV, while 22.1% experienced physical and emotional IPV, in the past year.

**Table 1:** Socio-demographics and IPV experience for sample (*n* = 935)

	*n*	%
Age 18/24	155	16.6
25/29	169	18.1
30/34	171	18.3
35/39	208	22.3
40+	232	24.8
Education None	778	83.5
Madrasa^a^	47	5
Primary	75	8.1
Secondary	32	3.4
Ethnic group: Pashtun	198	21.2
Tajik	500	53.5
Hazara	223	23.9
Other	14	1.5
Recent IPV: No emotional IPV	615	66.9
Emotional IPV	305	33.2
No physical IPV	716	76.8
Physical IPV	216	23.2
Recent IPV in three-level categorisation: None	597	66.2
One or more experiences of emotional IPV only	106	11.8
One or more experiences of physical IPV and one or more experiences of emotional IPV	199	22.1

^a^A religious school

There were low levels of education, with two-thirds having attended no schooling, and 13% only madrasa^1^ or primary school (Table 1). Women came primarily from three ethnic groups: the majority (53.5%) were Tajik, 21.2% were Pashtun, and 23.9% Hazara. A small proportion were from ethnic minority groups such as Uzbek or Turkmen.

IPV experience did not differ by age and education category (Table 2). A higher proportion of Pashtun women reported combined physical and emotional IPV compared to other ethnic groups.

**Table 2:** Descriptive associations between socio-demographic, and IPV risk factors for women experiencing past year emotional IPV, and emotional and physical IPV (*n* = 902)^1^

			No IPV		Emotional IPV only		Physical and Emotional IPV		
Demographic factors		*N*	%/mean	n/95%	%/mean	n/95%	%/mean	n/95%	*p*-value^3^
Age	Age 18/24	155	70.2	106	11.3	17	18.5	28	
	25/29	165	71.5	118	10.9	18	17.6	29	
	30/34	162	63.6	103	13.0	21	23.5	38	
	35/39	200	64.0	128	13.5	27	22.5	45	
	40+	224	63.4	142	10.3	23	26.3	59	0.5048
Education	Education: None	748	65.8	492	11.9	89	22.3	167	
	Madrasa^2^	46	58.7	27	10.9	5	30.4	14	
	Primary	74	66.2	49	14.9	11	18.9	14	
	Secondary	31	83.9	26	3.2	1	12.9	4	0.2845
Ethnic Group	Ethnic Group Pashtun	191	52.9	101	12.0	23	35.1	67	
	Tajik	482	72.4	349	10.4	50	17.2	83	
	Hazara	216	65.7	142	12.5	27	21.8	47	
	Other	13	38.5	5	46.2	6	15.4	2	< 0.00001
Individual level factors									
	Attend women’s group savings (no)	523	75.0	392	5.9	31	19.1	100	
	Attend women’s group savings (yes)	379	54.1	205	19.8	75	26.1	99	< 0.00001
	Individual gender attitudes	902	21.8	21.5–22.0	22.6	22.0–23.2	22.7	22.2–23.1	
	Childhood Trauma	900	14.8	14.6–15.0	15.8	15.0–16.5	16.4	15.8–17.0	
Livelihoods related factors									
	Food insecurity	901	4.7	4.5–4.9	6.2	5.7–6.7	6.6	6.2–7.0	
	Earnings past month	902	3382.0	3060–3705	3516.0	2673–4359	2603.0	2110–3096	
Family/husband factors									
	Only wife	838	68.5	574	11.5	96	20.1	168	
	More than one wife	63	34.9	22	15.9	10	49.2	31	< 0.00001
	Husband cruelty	902	9.8	9.7–9.8	9.9	9.8–10.0	11.1	10.9–11.2	
	Violence from other family member (no)	735	73.1	537	10.3	76	16.6	122	
	Violence from other family member (yes)	167	35.9	60	18.0	30	46.1	77	< 0.00001
	Perceived community gender attitudes	902	21.5	21.2–21.7	22.9	22.4–23.4	23.5	23.7–24.0	
Health factors									
	Depressive symptoms	901	12.5	11.9–13.1	15.8	14.1–17.5	20.8	19.3–22.4	
	Disability severity	897	1.0	0.9–1.1	1.7	1.4–2.0	2.4	2.2–2.7	

^1^ The difference in N between Table 1 and Table 2 is due to the categorization of IPV, where women (n = 17) who only experienced physical IPV were dropped from analysis; ^2^Madrasa – religious school; ^3^ *p*-values are calculated through chi-square tests for categorical variables

At the individual level, a higher percentage of women reporting emotional IPV, and those reporting physical and emotional IPV, reported being currently part of a savings group, or women’s group. Women reporting experiencing both physical and emotional IPV reported more inequitable gender attitudes and more experiences of childhood traumas, than those reporting no IPV, and the 95%CIs did not overlap. Women reporting emotional IPV only and physical and emotional IPV reported a higher mean score for food insecurity compared to those reporting no IPV, and 95%CIs did not overlap.

At the family level, a larger proportion of women reporting their husband had more than one wife, reported emotional IPV, and physical and emotional IPV. Women reporting physical and emotional IPV reported higher means scores for husband cruelty, than women with no IPV experience. And a higher proportion of women reporting emotional IPV, and physical and emotional IPV, reported experiencing violence from another family member. Women who experienced emotional IPV, and physical and emotional IPV, reported higher gender inequitable community attitudes, than those reporting no IPV. For health related factors, women reporting only emotional IPV, and physical and emotional IPV, reported more depressive symptoms, and higher mean scores for disability, than those reporting no IPV, with no overlap with 95%CIs.

In the multinomial regression (Table 3), women only experiencing emotional IPV, compared to no IPV, were more likely to be part of a women’s group and have higher household food insecurity. They were also more likely to report their husband had more than one wife, have experienced violence from another family member, and view community gender attitudes as less equitable.

**Table 3:** Multinomial regression for factors associated with emotional IPV, and physical and emotional IPV (*n* = 899)

	Past year emotional IPV only		Past year physical and emotional IPV	
Individual level factors	aRRR (95% CI)	*p*-value	aRRR (95% CI)	*p*-value
Education none	base		base	
Madrasa^1^	0.81(0.52–2.69)	0.711	1.20(0.43–3.35)	0.723
Primary	0.99(0.42–2.31)	0.974	1.29(0.56–2.96)	0.551
Secondary	0.24(0.03–2.00)	0.188	0.77(0.18–3.39)	0.732
Ethnic Group Pashtun	base		base	
Tajik	1.63(0.36–7.43)	0.531	0.65(0.15–2.77)	0.56
Hazara	0.36(0.06–2.39)	0.292	0.39(0.05–3.29)	0.388
Other	1.41(0.27–7.23)	0.684	0.28(0.03–2.93)	0.288
Currently attend a women’s group	**8.13(4.30–15.37)**	**< 0.0001**	**2.23(1.31–3.81)**	**0.003**
Individual gender attitudes	1.01(0.92–1.10)	0.91	1.03(0.95–1.12)	0.443
Childhood Trauma	1.04(0.96–1.12)	0.321	**1.10(1.02–1.18)**	**0.013**
Livelihoods				
Food insecurity	**1.13(1.03–1.25)**	**0.012**	1.06(0.97–1.16)	0.205
Earnings past month	1.00(1.00–1.00)	0.501	1.00(1.00–1.00)	0.629
Husband & family				
Husband has more than one wife	**4.22(1.66–10.73)**	**0.003**	**2.96(1.15–7.60)**	**0.024**
Husband cruelty	1.37(0.86–2.17)	0.188	**6.24(4.21–9.24)**	**0.001**
Experience of other form of family violence	**3.39(1.86–6.18)**	**< 0.0001**	**2.71(1.53–4.82)**	**0.001**
Perceived community gender attitudes	**1.13(1.02–1.25)**	**0.016**	**1.16(1.06–1.28)**	**0.002**
Depression	1.01(0.98–1.05)	0.465	1.01(0.97–1.04)	0.711
Disability severity	1.06(0.89–1.28)	0.503	**1.30(1.11–1.53)**	**0.001**

Adjusted for age, and cluster; all comparisons are to the category ‘none’

^1^A Madrasa is a religious school; aRRR – adjusted relative risk ratio

Bolded numbers of significant at *p* < 0.05

Experiencing physical and emotional IPV, compared to no IPV, was associated with participation in a women’s group, having higher levels of childhood trauma, reporting their husband had more than one wife, higher levels of husband cruelty, experiencing violence from another family member, and increased perceptions of gender inequitable attitudes at the community level. Women who reported greater levels of disability also reported more physical IPV and emotional IPV.

Table 4 shows the adjusted independent associations between experiences of IPV and health outcomes. Poorer life satisfaction was associated with women experiencing one or more emotional IPV episodes. Women reporting one or more physical IPV episodes and one or more emotional IPV episodes reported increased depressive symptoms, PTSD symptoms, worse life satisfaction, worse general health, increased suicidal ideation and greater severity of disability. Women reporting two or more emotional IPV episodes reported worse life satisfaction, worse general health, and higher levels of disability. Women reporting two or more physical IPV episodes and two or more emotional IPV episodes reported increased depressive symptoms, PTSD symptoms, worse life satisfaction, worse general health, increased suicidal ideation and greater severity of disability.

**Table 4:** Independent associations between IPV experiences and health outcomes

		Depressive symptoms		PTSD symptoms		Life satisfaction (> = less)		Health rating		Suicidal ideation		Disability severity	
IPV Categorisation (model 1)	*n*	adjusted ß coefficients	pvalue	adjusted ß coefficients	pvalue	adjusted ß coefficients	pvalue	adjusted ß coefficients	pvalue	aOR	pvalue	adjusted ß coefficients	*p* value
None	597	base		base		base		base		base		base	
One or more emotional IPV episodes only	106	0.48(−1.17–2.13)	0.569	0.07(−0.01–0.16)	0.096	**1.02(0.52–1.51)**	**< 0.0001**	0.14(−0.02–0.31)	0.087	1.60(0.53–4.79)	0.403	0.30(− 0.01–0.60)	0.059
One or more physical IPV and one or more emotional IPV episodes	199	**4.98(3.65–6.31)**	**< 0.0001**	**0.25(0.18–0.32)**	**< 0.0001**	**1.33(0.94–1.72)**	**< 0.0001**	**0.40(0.27–0.53)**	**< 0.0001**	**9.92(4.96–19.83)**	**< 0.0001**	**1.00(0.75–1.24)**	**< 0.0001**
IPV Categorisation (model 2)													
None	672	base		base		base		base		base		base	
Two or more emotional IPV episodes only	75	1.66(−0.23–3.54)	0.086	0.10(−0.00–1.20)	0.06	**1.09(0.53–1.66)**	**< 0.0001**	**0.32(0.13–0.51)**	**0.001**	1.17(0.32–4.23)	0.812	**0.48(0.13–0.83)**	**0.008**
Two or more physical IPV & two or more emotional IPV episodes	155	**5.39(3.97–6.80)**	**< 0.0001**	**0.23(0.15–3.03)**	**< 0.0001**	**1.18(0.75–1.60)**	**< 0.0001**	**0.43(0.29–057)**	**< 0.0001**	**8.49(4.52–15.94)**	**< 0.0001**	**0.99(0.72–1.25)**	**< 0.0001**

Adjusted for age, education, food insecurity and clustering

Bolded numbers are significant at p < 0.05

## Discussion

In our sample, about a quarter of currently married women reported any physical IPV in the past year, and just over a third reported emotional and/or physical IPV in the past year. Almost all those who experienced physical IPV, also experienced emotional IPV. Factors associated with women experiencing emotional IPV only, and physical IPV and emotional IPV, were very similar in the multinomial model, where typically women reporting physical and emotional IPV experience, compared to emotional IPV only, had larger adjusted relative-risk ratios.

Not all factors associated with women’s experience of emotional IPV, were associated with women’s experience of physical and emotional IPV. Specifically, food insecurity was only associated with experiencing only emotional IPV, but not physical and emotional IPV, and childhood traumas were only associated with experience of physical and emotional IPV, and not emotional IPV only. There are a number of potential reasons for this. Emotional IPV, while strongly overlapping with physical IPV, is a different construct [27], and as such may have slightly different drivers. Additionally, the relatively small sample size means that some of the adjusted risk ratios, while large, were not significant in the final model.

Women’s experience of both emotional IPV only, and physical IPV and emotional IPV, were associated with involvement in women’s groups outside the home. It may be women who experience IPV opt into women’s groups, more than women who do not experience IPV, as a way to build resources and social networks and work to ameliorate the impact of IPV, enabling resilience in the face of trauma [40]. Alternatively women’s participation in groups exacerbate women’s vulnerability to IPV, as they empower women and challenge the existing gender order [17]. However, analyses, including this one, do not establish the temporal order of these relationships.

Experience of childhood traumas were associated with experiencing physical IPV and emotional IPV, but not emotional IPV only. The importance of childhood traumas in shaping IPV experience is recognised in a variety of contexts [10]. Afghanistan has experienced 40 years of conflict, and much of the trauma experienced by these women as children, may be associated with war exposure [41], and the overlaps between childhood traumas and war trauma requires further investigation.

No markers of poverty were consistently associated with IPV. Food insecurity was only associated with emotional IPV in the multinomial model. There was no evidence that earnings had any association with IPV. This is in contrast to much research that suggests poverty is a driver of women’s experiences of IPV [22]. There are a number of potential explanations for his finding. It could be this was because there little variation in the sample, or that these measures of economic wellbeing fail to capture how poverty manifests itself in Afghanistan. However, the lack of association does question whether intervention working to strengthen women’s economic position on their own, can reduce IPV [40, 42].

The family structure and dynamics were important for women’s experience of IPV. Women reporting more husband cruelty, were more likely to experience physical and emotional IPV. This reflects the importance of male power in shaping women’s experiences of IPV [15, 43], and that IPV is an extension of men’s attempts to control women [7, 16]. Additionally, women who experienced violence from other family-members were more likely to experience emotional IPV, and physical and emotional IPV, suggesting a clustering of violence in the home. Research has highlighted clustering of mother-in-law violence [17, 19, 44], and violence against children [10, 45] with IPV, this analysis extends the range of perpetrators further, as we included siblings and fathers.

Women reporting their husband had more than one wife were more likely to experience emotional IPV, and physical and emotional IPV. Qualitative research suggests multiple wives living under one roof, may experience increased levels of competition between wives, around positioning in the household structure, and men may struggle to manage this dynamic, increasing conflict [46]. In addition, men may feel pressure for social reasons to enter into multiple marriages, exacerbating conflict with their wives [46].

The importance of findings about household structure and dynamics and its relationship to IPV, is it questions the narrow focus on dyadic models of IPV (i.e. violence between husband and wives). The assumption IPV is a dyadic phenomenon emerges from research in high-income countries, with a narrow interpretation of social relationships within the home. It does not recognise that in many households there are multiple generations and that gender and age hierarchies shape relationships, and power is contested between women, and not only between women and men.

Community level inequitable gender attitudes were associated with emotional IPV, and physical and emotional IPV. Studies elsewhere have shown that community level attitudes to gender and acceptability of IPV have an independent impact on women’s experiences of IPV [24]. Interventions working to transform community level gender attitudes are therefore critical to prevent IPV [47].

Severity of disability was a ‘driver’ of physical and emotional IPV and a consequence in this study. Disabled women globally experience increased vulnerability to IPV, because of gender inequalities, disability stigma and discrimination, and increased economic dependency on partners and caregivers [48, 49], but little research has focused on this in low- and middle-income countries. The impact of emotional and physical IPV on increasing the severity of disability was also highlighted (Table 4), emphasizing the bi-directional nature of this relationship. Further work to disentangle questions of severity, type of disability, and intersections with other forms of marginalization and IPV is important. In addition, developing approaches to ensure the inclusion of women with disabilities within IPV prevention programmes remains critical.

The analysis showed women’s experience of emotional IPV had an independent association with a range of health outcomes, particularly when it was severe (two or more episodes) emotional IPV. This supports the limited body of evidence that shows emotional IPV has an independent impact on women’s health, over and above physical IPV [29, 30]. Despite the growing evidence on the independent impacts of emotional IPV on women’s health, emotional IPV remains under-studied, and rarely considered within trial outcomes [30].

The study has limitations. Very few (*n* = 17) women had only experienced physical IPV and not emotional IPV, and these women were excluded from analysis. As data are cross-sectional temporal associations between factors are unclear. Women self-selected into the study, and therefore this is not generalizable to the population-level.

## Conclusion

We show a complex picture about the appropriate nature of interventions to prevent IPV in Afghanistan. In contrast to reviews showing the importance of economic empowerment interventions to reduce women’s experiences of IPV [40, 50], our findings provide weak support for this in Afghanistan. Markers of poverty, or wealth, were not associated with physical IPV, and only food insecurity was associated with recent emotional IPV. Moreover, women reporting involvement in groups outside the home were also more vulnerable to IPV, suggesting working with women in Afghanistan needs to be undertaken incredibly carefully. Our analysis highlighted the importance of husband cruelty, a clustering of violence in the household, and the importance of perceived community attitudes in driving IPV. This suggests economic interventions for women to prevent IPV, should also work with husbands, families and communities, if they are to prevent women’s experiences of IPV.

## Abbreviations

IPV: Intimate Partner Violence
PTSD: Post-traumatic stress disorders
WfWI: Women for Women International

## Footnotes

1 Madrasas (Islamic religious teaching centres) have a long history in Afghanistan and have traditionally focused on teaching the reading of the Koran and Islamic principles and morals, although other subjects such as literacy, numeracy, mathematics, geography, history, science, logic and literature have been included at different times in traditional and more modern historical moments.
